# Correspondence

**Published:** 1872-08

**Authors:** E. W. Lane

**Affiliations:** Scarboro’, Ga.


### CORRESPONDENCE.

SCARBORO', GA., July 19, 1872.

*Mess. Editors:* I have concluded to write you a few lines in regard to Bronchocele (or Goiter), as it prevails here.

There is a neighborhood on the head waters of Lott's Creek, containing about fifty families, and spread over a territory of about five by seven miles, the female portion of which seems to be predisposed to this disease. I have been acquainted with the place for thirty years, and have practiced there for fifteen years. There is rarely a female that has arrived at purberty that is not afflicted with it.—It has been so with almost all the women raised there. There never was a male known to have it in that settlement.

The disease yields readily to the iodine treatment. It is seldom that a physician is consulted in regard to it (so well do they understand the treatment). Any young woman moving there takes it; and those who are raised there and afterwards move out, get well without any treatment whatever. The disease is seldom seen in any other locality that comes under my knowledge. There are several creeks that have their head branches in that neighborhood. There are also many spring branches. The soil is sandy; growth principally pine, interspersed with broad-leaf blackjack. Growth on branches, black gum, pairm elder, white bay, titic, poplar and short-leaf pine. The drinking-water is obtained from wells, the average depth of which is about twenty feet. The habits and diet of the people are the same as in other places. Malarial fever seldom in that locality. Typhoid fever prevails there occasionally.

The locality is noted for its extreme healthfulness in other respects.

A great deal has been written and said on the disease in question. It seems not to be very well understood—at least its cause. I advance no theory, but simply state the facts.

Yours with great respect,

E. W. LANE, M. D.

